# Intelligent biosensors for human movement rehabilitation and intention recognition

**DOI:** 10.3389/fbioe.2025.1558529

**Published:** 2025-07-09

**Authors:** Mehrab Rafiq, Nouf Abdullah Almujally, Asaad Algarni, Mohammed Alshehri, Yahya AlQahtani, Ahmad Jalal, Hui Liu

**Affiliations:** ^1^ Department of Computer Science, Air University, Islamabad, Pakistan; ^2^ Department of Information Systems, College of Computer and Information Sciences, Princess Nourah bint Abdulrahman University, Riyadh, Saudi Arabia; ^3^ Department of Computer Sciences, Faculty of Computing and Information Technology, Northern Border University, Rafha, Saudi Arabia; ^4^ Department of Computer Science, King Khalid University, Abha, Saudi Arabia; ^5^ Department of Informatics and Computer Systems, King Khalid University, Abha, Saudi Arabia; ^6^ Department of Computer Science and Engineering, College of Informatics, Korea University, Seoul, Republic of Korea; ^7^ Guodian Nanjing Automation Co., Ltd., Nanjing, China; ^8^ Jiangsu Key Laboratory of Intelligent Medical Image Computing, School of Future Technology, Nanjing University of Information Science and technology, Nanjing, China; ^9^ Cognitive Systems Lab, University of Bremen, Bremen, Germany

**Keywords:** wearable sensors, remote sensing, rehabilitation, intelligent perception, human movement, motion intention recognition wearable sensors, motion intention recognition

## Abstract

**Introduction:**

Advancements in sensing technologies have enabled the integration of inertial sensors, such as accelerometers and gyroscopes, into everyday devices like smartphones and wearables. These sensors, initially intended to enhance device functionality, are now pivotal in applications such as Human Locomotion Recognition (HLR), with relevance in sports, healthcare, rehabilitation, and context-aware systems. This study presents a robust system for accurately recognizing human movement and localization characteristics using sensor data.

**Methods:**

Two datasets were used: the Extrasensory dataset and the KU-HAR dataset. The Extrasensory dataset includes multimodal sensor data (IMU, GPS, and audio) from 60 participants, while the KU-HAR dataset provides accelerometer and gyroscope data from 90 participants performing 18 distinct activities. Raw sensor signals were first denoised using a second-order Butterworth filter, and segmentation was performed using Hamming windows. Feature extraction included Skewness, Energy, Kurtosis, Linear Prediction Cepstral Coefficients (LPCC), and Dynamic Time Warping (DTW) for locomotion, as well as Step Count and Step Length for localization. Yeo-Johnson power transformation was employed to optimize the extracted features.

**Results:**

The proposed system achieved 90% accuracy on the Extrasensory dataset and 91% on the KU-HAR dataset. These results surpass the performance of several existing state-of-the-art methods. Statistical analysis and additional testing confirmed the robustness and generalization capabilities of the model across both datasets.

**Discussion:**

The developed system demonstrates strong performance in recognizing human locomotion and localization across different sensor environments, even when dealing with noisy data. Its effectiveness in real-world scenarios highlights its potential for integration into healthcare monitoring, physical rehabilitation, and intelligent wearable systems. The model's scalability and high accuracy support its applicability for deployment on embedded platforms in future implementations.

## 1 Introduction

New Intelligent Applications Emerging daily with each advancement in artificial intelligence, many intelligent applications are finding their way into the realm every day to make human life better. They include some applications in entertainment, medicine, indoor navigation, home automation, lifelogging, rescue, and surveillance (Qi et al., 2022; Wang et al., 2022; Yan et al., 2023; Iqra et al., 2025). Accessibility of the internet also drives the development of these applications primarily by letting developers access a huge sum of data. One example of such an application is the recognition and localization of human activity from data obtained from the IoT (Zhang et al., 2023a; Wen et al., 2023a; Wen et al., 2023b). Modern smart devices are equipped with multiple sensors to collect information about the locomotion and localization of a person (Li L. et al., 2023; Zhang et al., 2023k; Zhang et al., 2023l). If these smart devices are used correctly, their built-in sensors can accurately report users’ activities and locations. However, there is a significant challenge in processing sensor data because users are free to operate their smart devices in many different ways (Yao et al., 2023a). They could hold their device, mainly smartphones, in their hand or put them in their pockets or bags, which increases data sparsity and complicates the task. Besides, noise in the sensor signals is another challenge that needs to be addressed when processing IoT data. These sensors are very susceptible to interference (Zheng et al., 2022; Hu Z. et al., 2023; Zhou and Zhang, 2022; Zhang et al., 2022); sometimes, all the data can be corrupted, leading to misleading results for the artificial intelligence model. Such problems require innovative solutions that incorporate multisensory modalities, methods of feature selection, and advanced machine learning techniques. With the promise to revolutionize various aspects of our daily lives with the effective creation of accurate and reliable human locomotion recognition and localization systems (Zhao et al., 2022; Zhang et al., 2023m; Zhang et al., 2023n), this is a vital and exciting area of research.

The sensor modalities that are part of the IoT system in this study include smartphones and smartwatches. These devices provide data on accelerometers, gyroscopes, magnetometers, global positioning system (GPS), and microphone. The accelerometer senses the translational forces working on the smartphone in three axes, x, y, and z, thus estimating the speed and direction of movement (Zhang et al., 2023m; Zhang et al., 2023n). The gyroscope measures the orientation of the smartphone along the x, y, and z-axes, which can be used to estimate the orientation of the device. Simultaneously, the magnetometer provides information regarding the strength and direction of the earth’s magnetic field, which helps determine the absolute location of the user (Qu et al., 2023b). In addition, microphone data would be very useful in determining the location and activity that a user is performing. For instance, sound data may provide relevant information about the activity that is occurring such as heavy breathing of running or distinct sounds by the crowd in a shopping mall (Li et al., 2023c; Li Z. et al., 2023). Finally, the effectiveness of GPS in ascertaining a person’s location outdoors has been successfully established. However, GPS suffers from noise inside the building and its accuracy deteriorates. Instead of eliminating the GPS location, the other IoT sensors enhance it for better estimation of the user’s location (Qu et al., 2023a; Liu Z. et al., 2023; Liang et al., 2018). Inertial sensors comprise gyroscopes and accelerometers that are found in all types of devices, ranging from smartphones to smartwatches and many other wearable devices. One of the advantages of this sensor over camera-based methodologies and clear lines of sight methods is that it does not require any additional equipment or devices (Li H. et al., 2023). It also consumes power at a very low level, which renders it suitable for continuous and long-term monitoring purposes (Tigrini et al., 2024a). explored the feasibility of handwriting recognition using combined wrist and forearm EMG signals, confirming the potential of myoelectric control for intelligent human-computer interaction. The study employed consolidated machine learning techniques, including SVM, LDA, and KNN, along with advanced feature extraction from both time and frequency domains. Their findings highlight that integrating forearm and wrist EMG probes can significantly enhance handwriting recognition performance. Similarly (Tigrini et al., 2024b; Zhang et al., 2023o), proposed PHASOR, a phasor-based feature extraction method designed to enhance gait phase recognition using surface EMG signals. Achieving an accuracy of 82% in a five-phase gait classification task, PHASOR outperformed state-of-the-art deep learning approaches such as Rocket and Mini-Rocket, while also offering reduced computational time. The study also noted performance degradation when extending beyond the traditional stance and swing phase classification.

Two datasets are used for this study, KU-HAR and Extrasensory, to address the problems of noise, sparsity, and different user behaviors in IoT data. The KU-HAR dataset provides labeled examples of human activities recorded from wearable devices in a controlled setting, whereas the Extrasensory dataset provides extensive multi-modal sensor data gathered from an uncontrolled, real-world environment (Mekruksavanich and Jitpattanakul, 2021). These datasets provide a good starting point for developing a strong system for human activity recognition and localization by leveraging their various data features. However, GPS data provides critical spatial and temporal information that forms the basis of understanding and predicting human mobility and behavior under different circumstances (Yao et al., 2023a; Zheng et al., 2022; Hu et al., 2023b; Zhou et al., 2023). The use of GPS data and inertial sensors has a wide range of applications beyond HAR (Zhao et al., 2022; Zhu et al., 2023; Qu et al., 2023a). One of the physical behavior biometrics is gait analysis, which can be an innovative biometric authentication method that identifies individuals based on their unique walking patterns, known as gait (Qu et al., 2023b; Liu et al., 2023b). Similarly, the vast use of GPS data has changed many aspects of life, from location-based services to urban planning and transportation research (Liang et al., 2018; Liu et al., 2022; Ma et al., 2023).

However, some limitations prevent the proper exploitation of inertial sensors and GPS data for human activity recognition (Sikder and Al Nahid, 2021; Zhang et al., 2023p). Background noise, location variability, erratic human movement, and the wide variety of different activities make it difficult to achieve accurate recognition. This paper presents an advanced HAR and localization system that provides deeper insights into various forms of human locomotion using wearable sensors and smartphones (Dai et al., 2021; Bashar et al., 2020). Our methodology involves a Hamming window-based segmentation phase and an effective noise reduction process by using a second-order Butterworth filter. We outline our major contributions as follows.

• To compensate for the class imbalances observed in the Extrasensory dataset, the study employs the synthetic minority oversampling method (SMOTE) which effectively elevates model performance and robustness over rare performances.
• Enhancing the field’s understanding of spatial movement through innovative feature extraction and identification for localization tasks.
• A combination strategy that combines various machine learning and signal processing techniques to efficiently identify patterns of human activity.
• Comprehensive testing on three benchmark datasets shows that our solution outperforms the most cutting-edge methods.
• The robustness of the HAR model was enhanced by: Using data from several users and smartphones with varying ranges and using the human activity identification module of an indoor positioning system to enhance positioning results overall.

With the rapid proliferation of smart environments and ubiquitous computing, the demand for intelligent systems capable of understanding human behavior in real time has intensified (Wang W. et al., 2023). Human Activity Recognition (HAR) and localization systems not only play a vital role in healthcare monitoring, elderly assistance, and personal fitness tracking but are also becoming essential components in smart cities, intelligent transportation systems, and context-aware services. The growing integration of Internet of Things (IoT) devices in everyday life necessitates robust algorithms that can process vast, heterogeneous sensor data efficiently (Wang H. et al., 2023; Wang Q. et al., 2023). Moreover, the importance of real-time processing, scalability, and energy efficiency in such systems cannot be overstated, especially as the volume and variety of sensor data continue to grow. In this regard, combining traditional signal processing with machine learning techniques offers a powerful approach to managing complex data streams and extracting meaningful patterns. This study aims to contribute to this evolving domain by designing a hybrid system that addresses real-world challenges in activity recognition and localization through innovative preprocessing, feature extraction, and classification methods (Wu Y. et al., 2023; Wu et al., 2023b). By leveraging data from both controlled and uncontrolled environments, our system strives to bridge the gap between academic research and practical deployment.

The remaining content of the article forms the following parts. Section 2 describes a review of the literature on HAR using smartphone sensors. Section 3 elaborates on the proposed system design. Section 4 presents the experimental setup. A description of the results and the experimental data from this study is presented in Section 5. Section 6.1 introduces the implications of the proposed system, and Section 6 describes the conclusion and the next steps.

## 2 Literature review

The literature review explores various approaches to recognizing human locomotion using sensory data. Studies have examined methods such as thermal imaging combined with generative adversarial networks (GANs) for identifying joint and skeletal information, as well as deep learning models like CNN and LSTM to classify human activities (Wu et al., 2023c; Xu Y. et al., 2023). Research also highlights the use of inertial sensors, particularly in smartphones, to track movement and classify actions like walking and stair climbing. Furthermore, advancements in multisensory systems, integrating data from GPS, IMUs, and ambient sensors, have improved the accuracy of locomotion recognition by addressing challenges like noise and irregular mobility patterns (Xu F. et al., 2023; Xu G. et al., 2023; Yang et al., 2023). These systems, leveraging techniques like segmentation, noise reduction, and feature extraction, are pivotal in applications such as healthcare and sports analytics.

### 2.1 Visual sensory-based recognition of human locomotion

A study introduced a new technique for gleaning details about joints and skeletons from photographs (Batchuluun et al., 2021). First, a single-channel thermal image was converted to a three-channel image. The photographs were merged in this way to enhance the information extraction process. In the study, a generative adversarial network (GAN) was used to help extract skeletal and joint data. Moreover, with the information obtained about skeletons and joints, the study attempted to identify various human gestures. CNN and LSTM were the two methods applied in combination to identify human activities. When the study tested their approach using both publicly available data and data they had individually collected, they found that it performed well compared to other best practices. However, the performance of the system is poor due to its inability to recognize images with limited spatial textual information. The study developed a model to recognize various human behaviors in a real-time healthcare environment (Yin et al., 2021). The authors used the multichannel LSTM. This system was developed to recognize activities using three-dimensional skeleton data. A unique loss function was added to enhance accuracy. They used two benchmark datasets: the TST fall detection database and the NTU RGB + D dataset. However, the capacity of the system to deliver skeleton data flawlessly is constrained because it uses a frame-level error detection methodology. It fails to identify the cause of dimensionality-related problems and, as a result, compromises the overall accuracy of the system. Using different video frames, the authors of a different study (Cheng et al., 2023) concentrated on activity recognition. A second spatial attention module and residual CNN are used to identify activities. The performance of the proposed system suffers from the absence of integrated optical flow maps. To track human motion, recent research has advanced remote sensing techniques and concentrated on developing effective traffic monitoring systems. Human locomotion activity recognition (HLAR) relies heavily on inertial sensors, particularly those found in smartphones (such as gyroscopes and accelerometers). The study by (Xie et al., 2018), for example, employed these sensors to extract characteristics, classify them using deep neural networks, and select them using neighborhood component analysis. To identify actions such as walking and stair climbing, (Xie et al., 2017; Lee et al., 2017), investigated kernel functions in an SVM model and verified their findings using 10-fold cross-validation.

Hsu et al. (Abdel-Basset et al., 2022; Konak et al., 2016) integrated CNN with LSTM for sensor data classification improvement, whereas (Chetty et al., 2016) used wearable inertial sensors to track the movements of the body. A different study (Ehatisham-ul-Haq et al., 2020; Azam et al., 2020) used naive Bayes classifiers, decision trees, and random forests to categorize activities. Its small sample size, however, raised concerns about its generalizability. Our approach, on the other hand, uses hybrid LSTM and the Extrasensory dataset (60 participants) to produce a more reliable model.

The information theory-based feature ranking algorithm created by (Chetty et al., 2020; Mutegeki and Han, 2020; Han et al., 2020) was only evaluated on one dataset, which limited its practical use. Our model improves adaptability after being trained on a variety of datasets. Although multimodal inputs were combined by (Ehatisham-ul-Haq et al., 2022; Liu et al., 2022) for robust activity recognition, our hybrid LSTM performs better in challenging tasks. For simple jobs, Mutegeki et al. (Jaramillo et al., 2022) employed CNN-LSTM, but they had trouble performing more complicated operations. While (Hussain et al., 2023) used five deep-learning architectures to identify human activities, they encountered lengthy training times (Hajjej et al., 2023; Garcia-Gonzalez et al., 2023). used Quaternion-based filtering and data windows. On the other hand, our method attains great accuracy in fewer epochs. Lastly, HAR was developed using EEG and smartphone sensor data by (Zhang et al., 2021; Al-qaness et al., 2022a) but with limitations due to dataset homogeneity.

### 2.2 Wearable system recognition of human locomotion

(Mutegeki and Han, 2020; Han et al., 2020) proposed an integrative architecture of deep learning for recognizing activity by a CNN-LSTM model. In addition to reducing model complexity and, in this case, ending the need for intricate feature engineering, the approach purported to improve predictability related to human activity from raw data. The network architecture CNNLSTM required deep temporal as well as spatial dimensions that were proposed. The model had 99% and 92% accuracy rates when tested on the publicly available UCI HAR dataset and the internal iSPL dataset. Results, however, indicate degradation of performance when handling complex activities like atomic-level actions. Additionally, the SoftMax loss increased as the model complexity increased, indicating that combining CNN and LSTM layers did not improve the results (Jaramillo et al., 2022). used an approach known as quaternion filtration in a system with one sensor. In the next step, several segmentation methods were used to divide the data. Features are then eliminated. Finally, activities have been classified using the LSTM classifier. We found that the system requires more processing power.

As per (Hu F. et al., 2023), an IMU sensor-based human activity detection system uses a data set generated through wearable devices. Many preprocessing operations are used, including moving averages, sliding overlap windows, and data segmentation. CNN, recurrent neural network, LSTM, bidirectional LSTM (BiLSTM), and gate recurrent unit are the five different classifiers utilized for activity recognition. The high number of epochs in the proposed system makes it especially costly in terms of temporal complexity. The hidden Markov model is a relatively new concept among researchers (Liu et al., 2018; Schultz et al., 2018). The ability to logically model a time series gives the recognition of human activity some interpretability.

### 2.3 Multisensory system recognition of human locomotion

Multisensory systems integrate data from various sensors, such as inertial measurement units (IMUs), GPS, and ambient sensors, to enhance human locomotion recognition accuracy. These systems leverage the complementary nature of different sensor modalities to overcome challenges such as noise, diversity of activities, and irregular human mobility patterns. For instance, GPS sensors provide spatial-temporal data critical for localization tasks, while IMUs like accelerometers and gyroscopes offer precise information about movement dynamics. The study (Yin et al., 2021) developed a model to detect different human actions in a real-time healthcare environment. The authors utilized a multichannel LSTM.

Recent advancements in signal processing and machine learning have enabled the development of robust multi-sensory frameworks. Techniques such as segmentation using Hamming windows, noise reduction via Butterworth filters, and feature extraction methods like skewness, kurtosis, and LPCC enhance data reliability and interpretability. Furthermore, integrating classifiers such as CNN-LSTM or Fuzzy Entropy classifiers has improved activity recognition performance, even for complex locomotion tasks. Such systems are instrumental in applications like health monitoring, sports analytics, and navigation. By optimizing features using methods like Yeo-Johnson Power Optimization, multisensory systems can provide reliable and efficient solutions for recognizing diverse locomotion behaviors across varied environments.

## 3 Materials and methods

**FIGURE 1:**
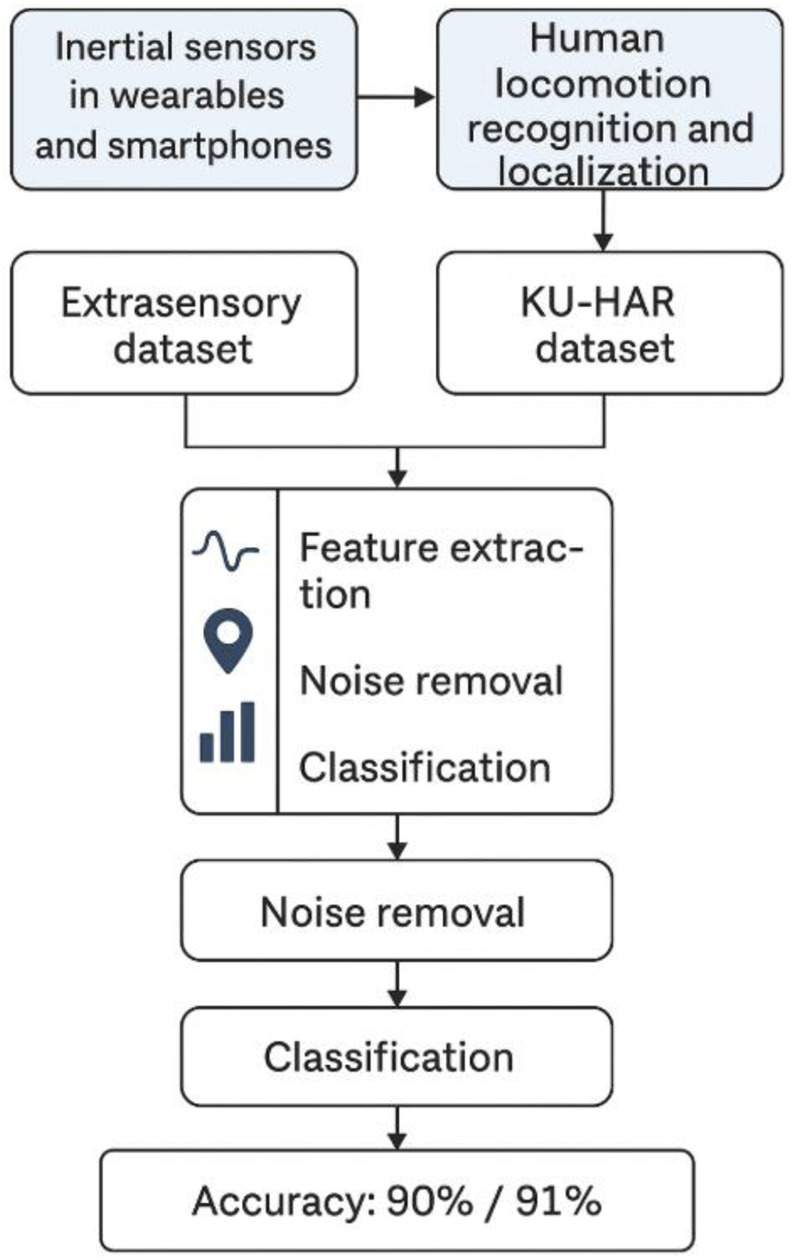
The graphical abstract of the system.

This section describes, in detail, the recommended Human Locomotion and Localization process. To address this problem, we proposed the following methodology (Figure 1):

To overcome the issues, the architecture is rigid and well-defined. The second-order Butterworth filter is used to pre-process the input signal to enhance quality and reduce noise. Hammering windows are used to divide the pre-processed signal for windowing. Localization (Step Count and Step Length) and locomotion (Linear Prediction Cepstral Coefficient (LPCC), Dynamic Time Wrapping (DTW), Skewness, Kurtosis, and Energy) make up feature extraction. The Fuzzy Entropy Classifier is used for classification, and Yeo John Power Optimization enhances features.

### 3.1 Preprocessing

**FIGURE 2:**
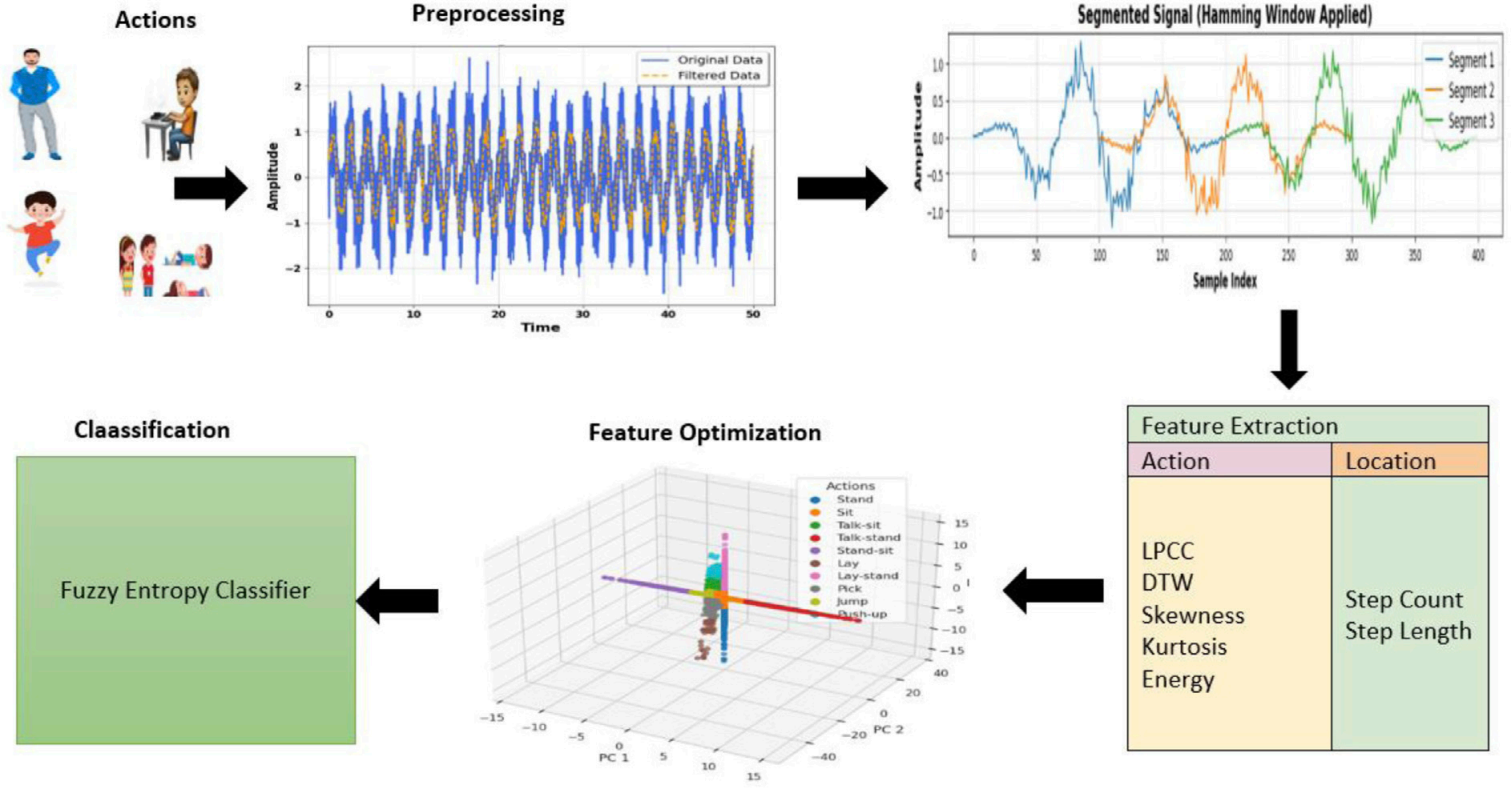
The architecture of the proposed system.

Preprocessing is an important data analysis step, ensuring the quality and reliability of data when further analyzed and features extracted. It cleans, transforms, and enhances raw data to reduce noise and clear the signal (Yao et al., 2023b; Zhang et al., 2023b). Among these techniques, filtering is widely used in preprocessing, mainly to remove unwanted components that may be present in data, such as high-frequency noise (Tayyab and Jalal, 2025). This filter is widely used because it has a flat frequency response in the passband and is ideal for signal preservation while removing noise. The Butterworth filter of order two is a good balance between computational efficiency and performance in terms of the smooth transition from the passband to the stopband (Mahmood et al., 2020; Azmat and Ahmad, 2021.). This filter is defined by its transfer function, which ensures minimal distortion to the original signal. It is the second-order Butterworth filter as preprocessing in both graphs. Specifically, the preprocessing is applied on the KU-HAR dataset, as demonstrated in Figure 1, and on the Extrasensory dataset (Zhang et al., 2023c). The preprocessing using the second-order Butterworth filter as in shown in equation 1 which effectively removed the noise while keeping the intrinsic characteristics of the data that ensured clarity and reliability in further analysis (Muneeb et al., 2023). These preprocessing steps are important in preparing the datasets for subsequent feature extraction and visualization (Mahwish and Ahmad, 2023), thereby enhancing the quality of the results and their ease of interpretability (See Figure 2).

$$\left|H\left(w\right)\right|=\frac{1}{\sqrt{1+\in^{2}T_{n}^{2}\;\left(\frac{\omega }{\omega_{{}^{\circ}}}\right)}}$$ (1)

### 3.2 Window and segmentation

**FIGURE 3:**
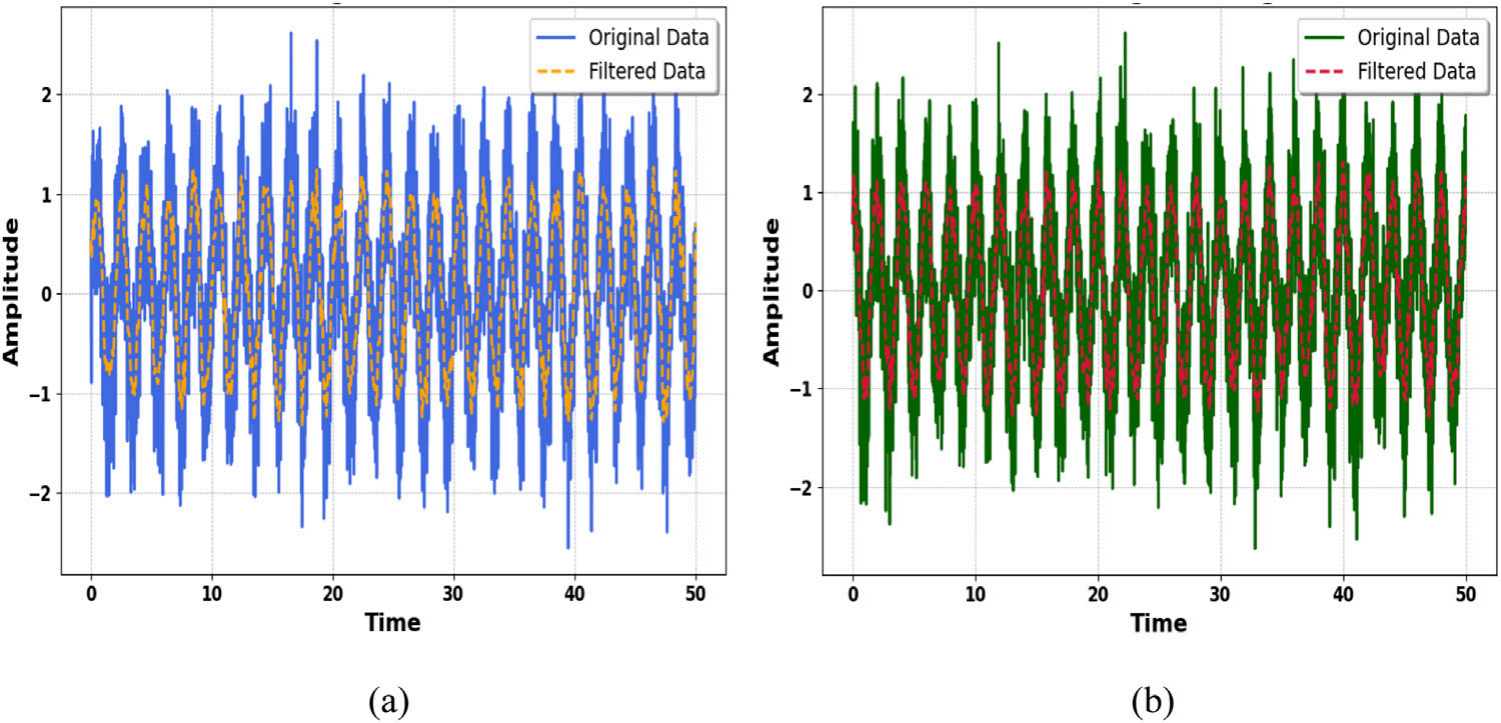
Pre-processing using second order Butterworth filter **(a)** KU-HAR Dataset **(b)** Extrasensory dataset.

Windowing and segmentation are important steps of time series data analysis, particularly in applications such as signal processing and activity recognition (Zhang et al., 2023b). The idea of these techniques is the division of continuous data into a smaller number of overlapping, or non-overlapping pieces, called windows. Segmentation simplifies complex data streams, allowing for localized analysis and extraction of relevant features from each segment, which is very important for pattern identification and model improvement (Zhang et al., 2023d). In this research, the Hamming window was used during the windowing process. The Hamming window is a tapered window function that minimizes spectral leakage by smoothly reducing the signal amplitude at the edges of the window while preserving the central portion of the data (Majid et al., 2020). This property ensures that each segment captures the key characteristics of the signal without introducing artifacts, thus improving the reliability of subsequent feature extraction and classification tasks (Saleha et al., 2025). The application of the Hamming window to the segmentation process improved the quality and resolution of the data, thus making it more suitable for accurate analysis and modeling is shown in Figure 3. The hamming window is implemented using Equation 2.

$$\omega \left(x\right)=0.54-0.46\cos\left(\frac{2\pi x}{X-1}\right)$$ (2)

where Wn is the value of the hamming window at index n, while N is the length of the window.

### 3.3 Feature extraction for locomotion’s

It has two sections on feature extraction: one deals with localization, and the other with movement. The steps count and the length of steps (localization) as well as locomotion belong to the feature extraction such as LPCC, DTW, skewness, kurtosis, and energy. Classification is by Fuzzy Entropy Classifier, while Yeo John Power Optimization enhances the feature.

#### 3.3.1 Linear prediction cepstral coefficient (LPCC)

**FIGURE 4:**
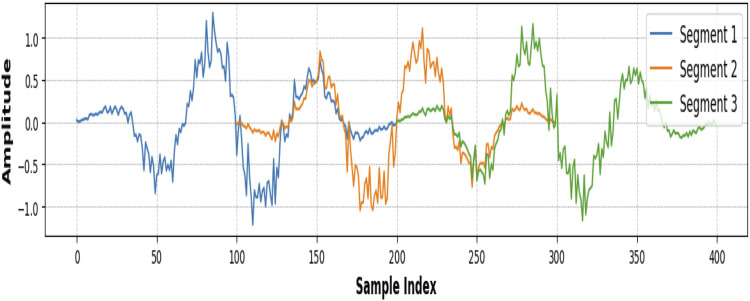
Windows and segmentation using the Hamming window.

For the task of feature extraction, Linear Prediction Cepstral Coefficients (LPCC) were used because they are robust in capturing spectral characteristics of time-series signals. LPCCs are based on the linear predictive coding model that predicts future samples of a signal from its past values. The cepstral coefficients represent the logarithmic spectrum of the signal (Fatima et al., 2024a), hence capturing its fundamental features in the frequency domain. Features were extracted using LPCCs from the KU HAR dataset as shown in Figure 1. In human activity recognition, LPCCs were used to classify the activities (Iqra and Jalal, 2025). They picked up the unique spectral pattern relating to the different activities undertaken during their execution, such as walking or jumping while sitting. The compact and discriminative representation improves the classification performance of features obtained from the underlying activities. This scenario happened to be the case for Extrasensory as well. This dataset includes a wide variety of sensory data, which contains motion and environmental signals where LPCCs captured the subtle variations in the signal spectrum across different activities and contexts. The application of LPCC to both datasets demonstrated its versatility and effectiveness in representing temporal and spectral characteristics, facilitating accurate and reliable activity recognition in diverse scenarios (See Figure 4). The LPCC is implemented using Equation 3.

$$x\left(n\right)=-\sum_{k=1}^{p}a_{k}x\left(n-k\right)+e\left(\;n\right)$$ (3)

x(n) is the current sample of the signal at time n, $a_{k}$ ​ are the LPC coefficients, p is the order of the predictor, x(n−k) is the past samples, and e(n) is the prediction error (or residual).

#### 3.3.2 Dynamic time wrapping (DTW)

**FIGURE 5:**
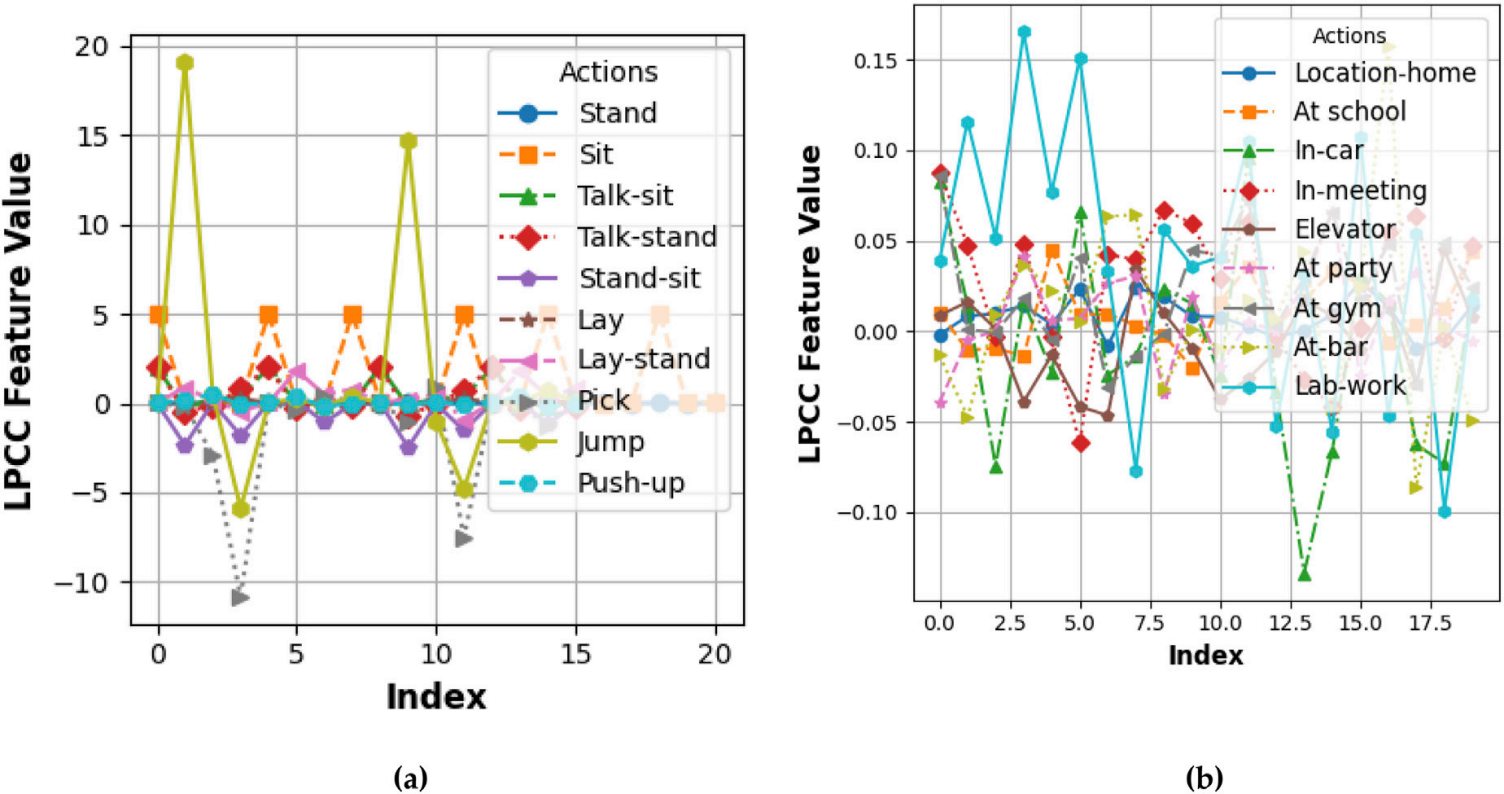
LPCC computed for different locomotion over **(a)** the KU-HAR dataset and **(b)** the Extrasensory dataset.

DTW is one of the widely used feature extraction techniques and similarity measurement in time-series data. It has its best applications in comparing sequences that differ in speed or length. The basic idea behind DTW is to warp the time axes of two time-series sequences so that the distance between them is minimized (Amir et al., 2020). This alignment allows for a robust comparison by accounting for variations over time, making DTW an ideal algorithm for the analysis of time-dependent data such as human activity recognition. DTW was applied to the KU-HAR dataset for analyzing and extracting features from various human activities. The dataset contains time-series data representing actions such as walking, sitting, standing, and jumping (Seerat et al., 2025). The DTW distance and alignment of activity sequences captured both temporal and structural properties of actions and were used for calculating them. This led to discriminative feature extraction that made it possible to clearly explain the relation of activities based on time-series patterns between different activities. By using DTW, this feature extraction process captures the variability in human movements effectively and provides a robust representation towards subsequent classification and analysis processes (Zhang R. et al., 2023). This approach demonstrates its aptness to address real variations in activity execution within real application scenarios, which is shown in Figure 5. DTW is calculated using Equation 4.

$$D\left(i,j\right)=d(x_{i},y_{i})+\min\left\{\begin{array}{l} D\left(i-1,j\right),\left(insertion\right)\\ D\left(i,j-1\right),\left(deletion\right)\\ D\left(i-1,j-1\right),\left(match\right) \end{array}\right.$$ (4)

#### 3.3.3 Skewness

**FIGURE 6:**
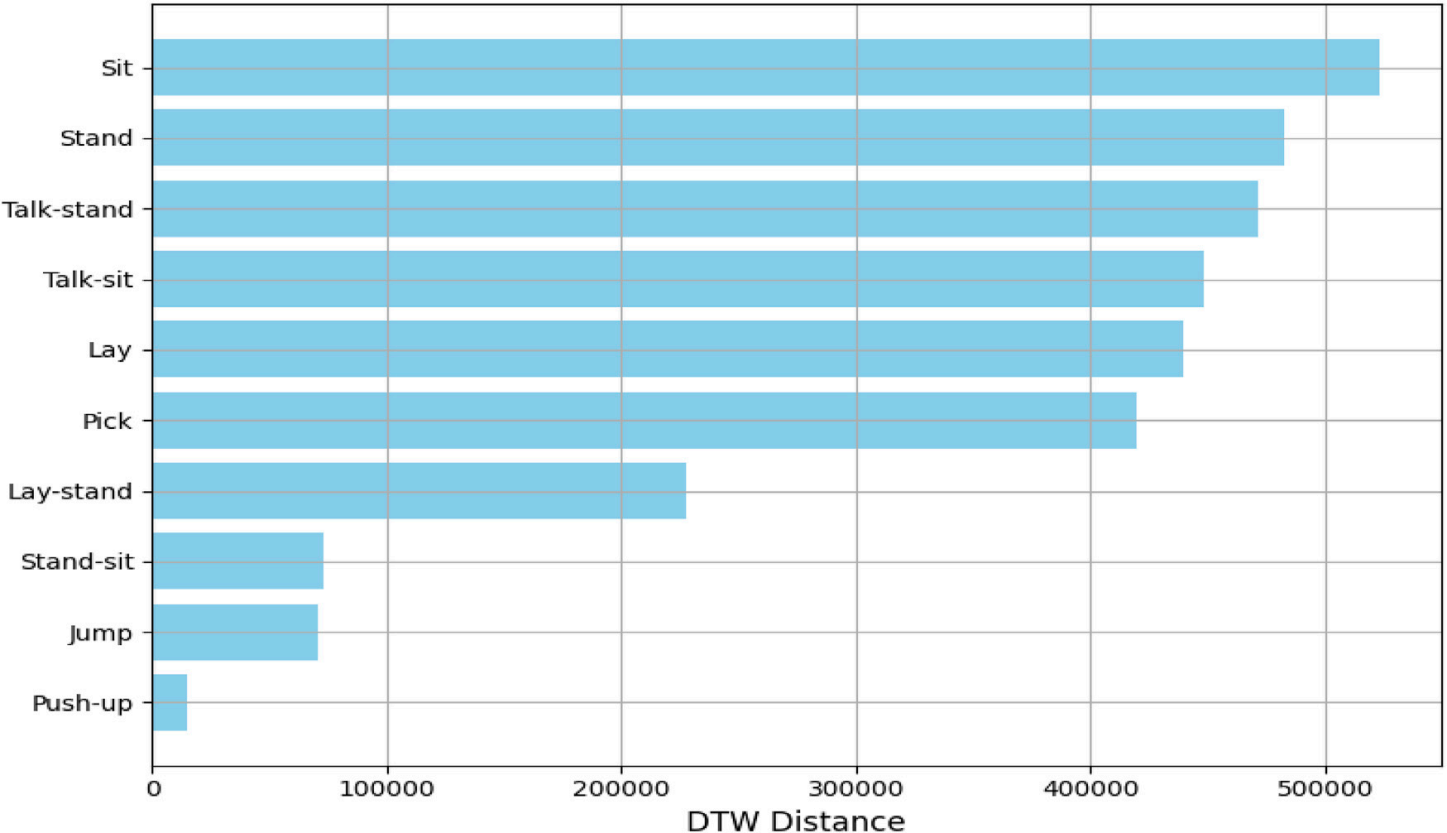
DTW computed for different locomotion over the KU-HAR dataset.

Skewness is a statistical measure that describes the asymmetry of distribution of probabilities of a dataset relative to its mean. In such a manner, it offers valuable insights into the shape of the data distribution. Positive skewness represents a long right tail; negative skewness symbolizes a long left tail of the data distribution. Skewness is used in feature extraction to capture the asymmetry of time-series signals, providing a discriminative characteristic to distinguish between different patterns or actions (Ahmad et al., 2019). Skewness was calculated as a feature from the KU-HAR dataset, which contains time-series data of various human activities such as walking, sitting, standing, and jumping. The skewness values of these actions were analyzed to identify their distinct distribution patterns (Hafeez et al., 2021). A graph has thus been designed to portray different activities to visualize their respective skewness (Zhang et al., 2023f). Based on the different skewness measures of different activities, its variation from asymmetrically of the data is reported. Hence, for skewness features, a special distinction profile in the skewness could always be there for differentiation classification. As such, capturing different variations of signal distributions might make a feature more feasible in recognizing human activities while leveraging the effectiveness of these extracted features. This method thus proved the use of skewness in describing and distinguishing complex time-series data (See Figure 6). Skewness is calculated using Equation 5.

$$Skewness=\frac{n\;\sqrt{n-1}}{n-2}\frac{\sum_{i=1}^{n}\left.(x_{i}-\overset{\ldots }{\left.x\right)}\right.}{s^{3}}$$ (5)

#### 3.3.4 Kurtosis

**FIGURE 7:**
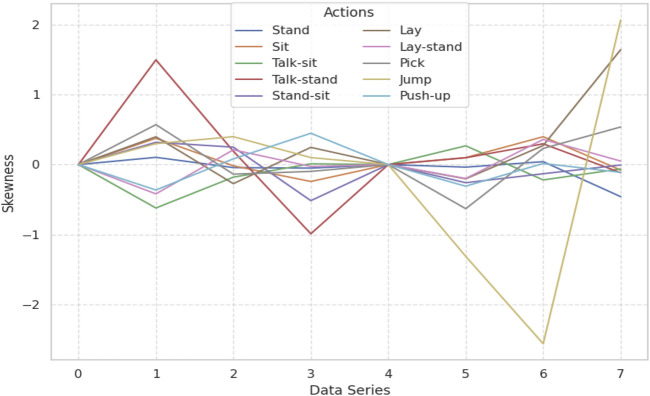
Skewness calculated over the KU-HAR dataset.

Kurtosis is a statistical measure that quantifies the “tailedness” or sharpness of a data distribution relative to a normal distribution. It gives information about the extremities of the data and whether the distribution has heavier or lighter tails compared to a normal distribution (Zahra et al., 2025). A high kurtosis value indicates that the distribution is peaked with heavy tails, while a low kurtosis indicates flat distributions. In feature extraction, kurtosis is used in the process of analyzing the shape of time-series data. Such analysis captures crucial characteristics, which distinguish activities or patterns (Zhang T. et al., 2023). Kurtosis was determined for the KU HAR dataset, which contained time-series data representing several human actions, including walking, sitting, standing, and jumping. A graph was constructed to illustrate the values of kurtosis for various activities, demonstrating the difference in the shape of the distribution of data between these different activities (Iqra and Ahmad, 2024a). The kurtosis graph showed clear patterns for each action, which was an indication of the differences in their data distribution characteristics (Tayyab et al., 2025). Actions with sharper peaks and heavier tails were more kurtosis, while actions with flatter distributions were less kurtosis. By including kurtosis in feature extraction, this work captured critical aspects of the data distribution, thereby enhancing the differentiation and classification of human activities. The graphic displayed a clear visualization of such patterns so that it could better explain the peculiar characteristics of each action (See Figure 7). Kurtosis is calculated using Equation 6.

$$Kurtosis=\frac{n\left(n+1\right)}{\left(n-1)\left(n-2\right)\left(n-3\right)\right.}\sum_{i=1}^{n}{\left(\frac{x_{i}-\bar{x}}{s}\right)}^{4}-\frac{3{\left(n-1\right)}^{2}}{\left(n-2\right)\left(n-3\right)}$$ (6)

#### 3.3.5 Energy

**FIGURE 8:**
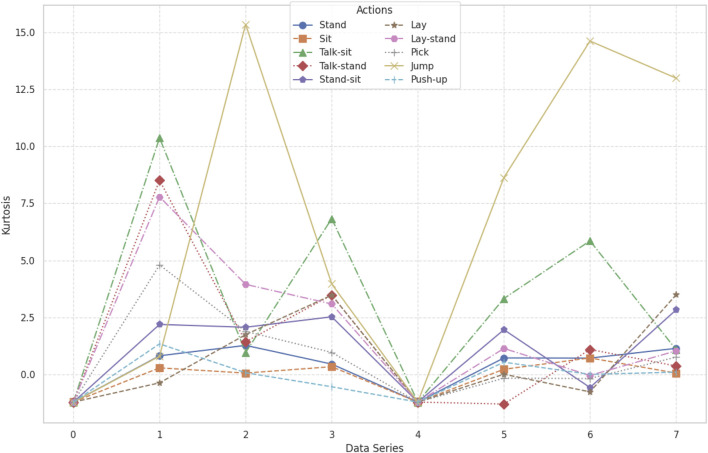
Kurtosis calculated over the KU-HAR dataset.

Energy is one of the features widely applied in time-series analysis and signal processing. It measures the sum of the squared magnitude of the signal over a window or segment. This will give the total power or intensity of the signal (Iqra and Ahmad, 2024b). It is used as a robust measure for activity recognition or pattern identification between various activities. It is one of the features used for human activity data because energy shows the difference in intensity of the signal, related to different movements. The KU HAR dataset involves time-series data for walking, sitting, standing, and jumping activities. The energy level associated with each activity was graphed to visualize it. Patterns in the energy graph were unique, with large energy values for jumping, which has high energy, while sitting activity had low-energy values (Zhang F. et al., 2023; Naif et al., 2025). This visualization gave insights into the intensity of each action and its variability over time. By including energy as a feature, the study captured dynamic differences in activity patterns, allowing for better classification and analysis (Laiba and Ahmad, 2024a). The energy graph served as a powerful tool to interpret the data, bringing out the unique characteristics of each activity in terms of signal intensity as shown in Figure 8. Energy function is calculated using Equation 7.

$$E=\int_{-\infty }^{\infty }{\left|x\left(t\right)\right|}^{2}dt$$ (7)

|x(t)| is the magnitude of the signal at time t.

### 3.4 Feature extraction for location

#### 3.4.1 Step count

Step count is the number of steps taken during a certain period in human activity recognition and behavior analysis. It is a critical feature often derived from accelerometer data where periodic peaks in signal magnitude correspond to individual steps (Fatima et al., 2024b). Step count is most useful for feature extraction as it can provide insight into physical activities, such as walking or running, or transitions between an active and a sedentary state. In the chart Extrasensory dataset applies for a particular location, index, or time progression that appears on the x-axis and magnitude, which is its proxy for step count on the y-axis (Hanzla and Jalal, 2025). The vector sum of the three axes of the accelerometer computes as the signal magnitude: This graph is divided into two phases, training data (blue), and validation data (orange). Periodic fluctuations in the signal correspond to steps, peaks of which correspond to the moments of increased acceleration caused by footfalls (Zhang et al., 2023i). The part between indices 0–2,000 (validation data) demonstrates a steady activity so it can be a walk or run, whereas the rest of the training data is more varied and can correspond to transitions between different activities or periods of rest as shown in Equation 8.

$$L=k.\;\sqrt{H.∆a_{stride}}$$ (8)

where: k: Calibration constant (accounts for individual differences), h: Height of the individual (correlated with step length), $∆a_{\text{stride}}$ : Average acceleration during one stride, calculated as the difference between peak and trough acceleration for each step.

**FIGURE 9:**
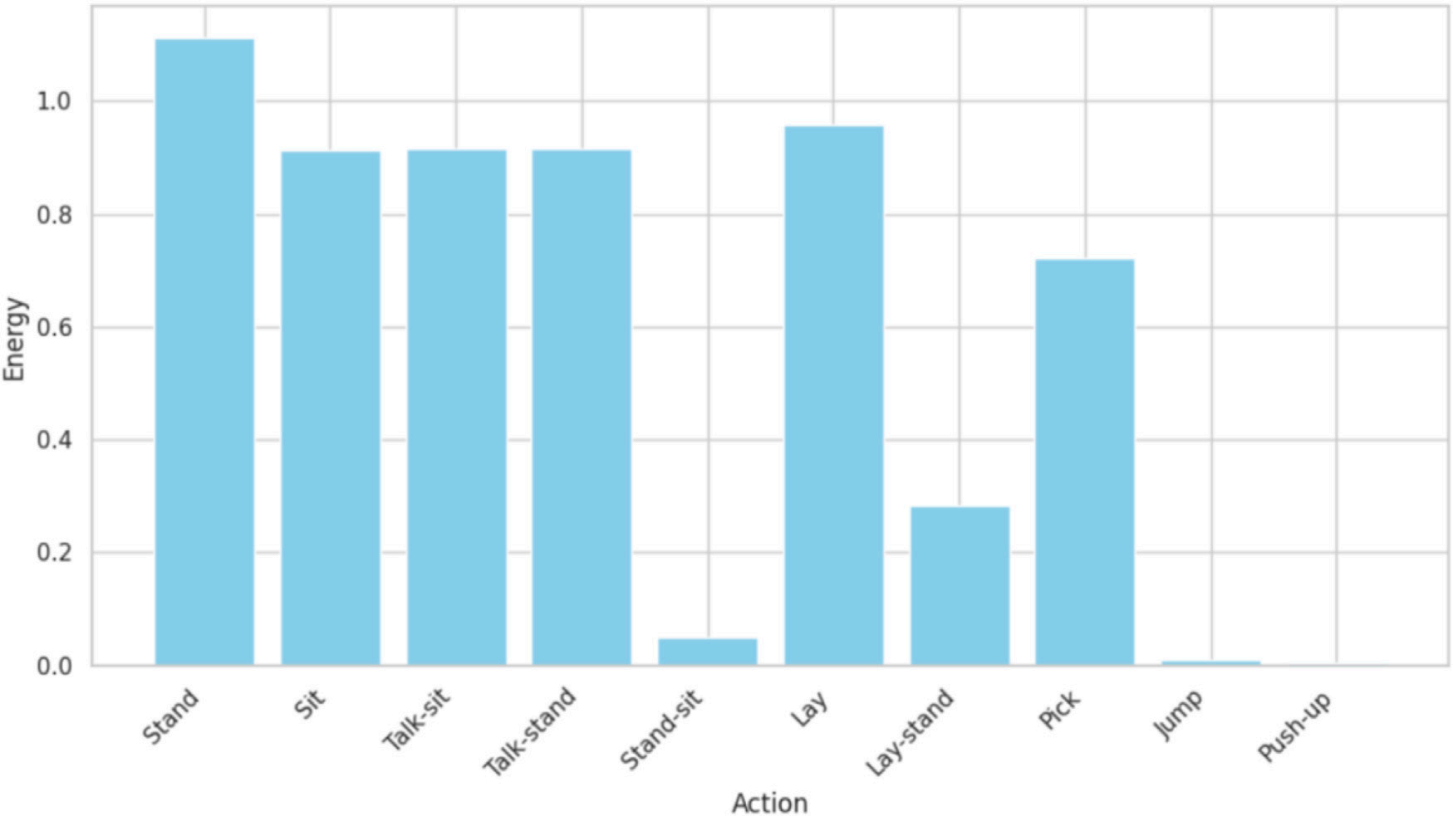
Energy calculated over the KU-HAR dataset.

For feature extraction, the step count can be quantified by detecting these peaks in the magnitude signal. Other features that can be derived from the step count data include the total number of steps, step rate, statistical measures such as mean, variance, and standard deviation, and energy of the signal. These features are invaluable for recognizing patterns, analyzing activity levels, and understanding user behavior in different locations or contexts. From the graph, you can identify activity trends and transitions that serve as a basis for developing robust activity recognition systems by visualizing step counts (See Figure 9).

#### 3.4.2 Step length

**FIGURE 10:**
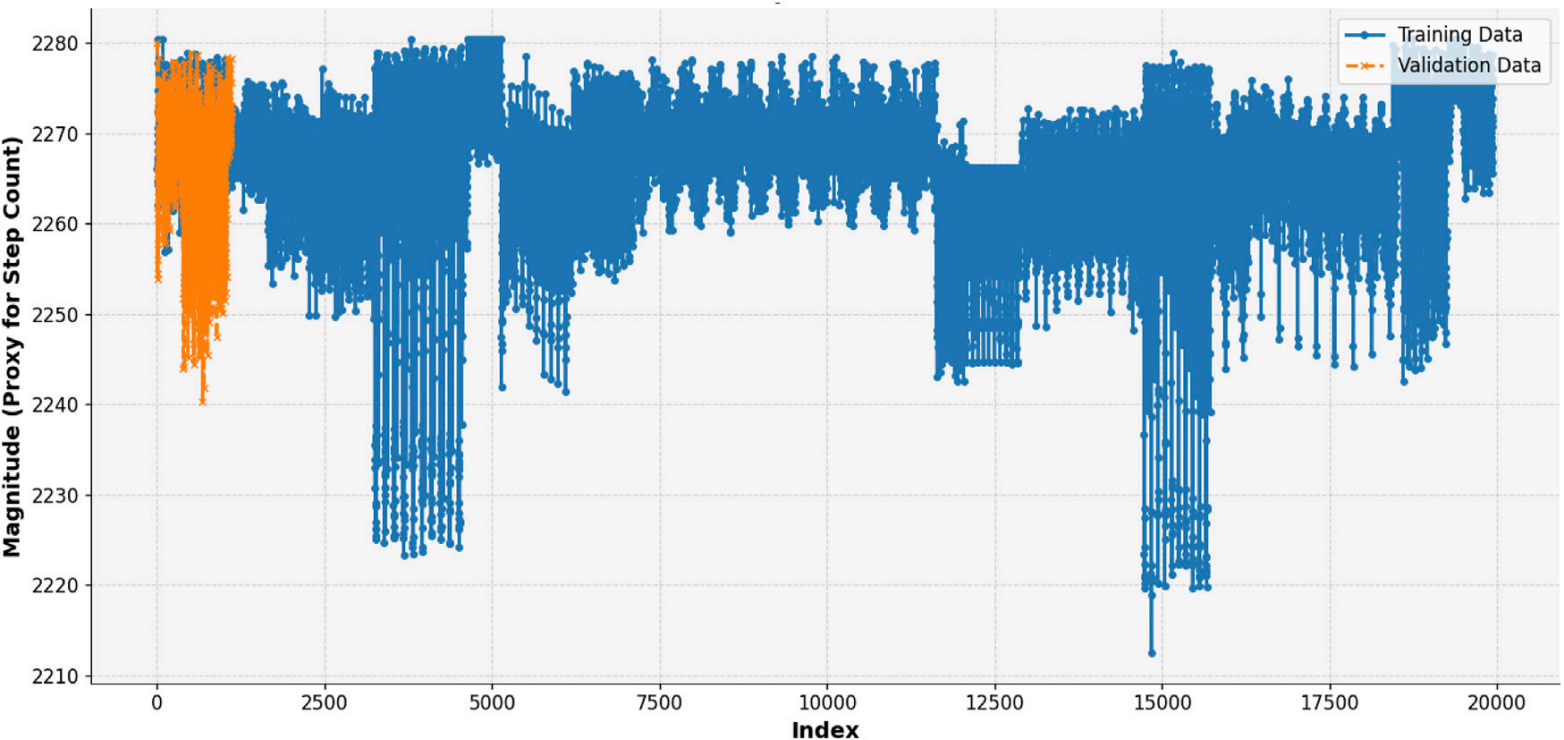
Step count calculated over the KU-HAR dataset.

The distance traveled in one step is represented by the step length feature, which is important in the analysis of human mobility (Ahmad et al., 2025). It can be helpful to understand the characteristics of movement, such as walking or running, and offers insight into the person’s gait and stride. The environment or location also influences step length since it depends on factors like walking speed, terrain, and individual behavior for covering a distance per step (Sumbul et al., 2025). The Extrasensory dataset, which contains sensor data from various sources such as accelerometers and location-tracking sensors, was used to calculate step length. This dataset contains both activity and location information, allowing us to estimate step length across different locations (Hanzla et al., 2024). The sensor signals were analyzed to derive step length by detecting the peaks corresponding to each step and measuring the distance between them (Ashraf et al., 2025). A graph was plotted to show step length for different locations in the dataset. This gives an idea of how movement changes in different environments. For instance, outdoor locations or open spaces would have longer step lengths because one would walk at a normal pace, whereas indoor locations or constrained spaces would have smaller step lengths due to a lack of space or reduced movement. The graph provided a clear comparison of step length across the locations, showing how spatial context influences the individual’s movement (Saleha and Jalal, 2024; Sara et al., 2024). This study thereby extracted and visualized the step length for different locations, which brought to the fore the relationship between the movement patterns and the environment. The step length graph was a powerful tool that helped understand the dynamics of human movement across different spaces, aiding in location-based activity recognition (Zhang et al., 2023j; Nazar and Jalal, 2025). This would enhance the ability to distinguish between activities and locations in the context of mobility characteristics. The importance of this feature for the enhancement of location-aware system accuracy, as well as insights into how environmental factors may impact step length and movement patterns, cannot be overlooked, is shown in Figure 10. Step length is calculated using Equation 9.

$$S=\frac{\sum_{i=1}^{n}\left|a_{i}\right|}{∆a_{threshold}}$$ (9)

where: $a_{i}$ : Accelerometer data at time i (magnitude of acceleration vector: $\sqrt{{x_{i}}^{2}+{y_{i}}^{2}+{z_{i}}^{2}}$ ), $∆a_{\text{threshold}}$ : A threshold value to detect step peaks (based on signal filtering and gait analysis), n: Total number of samples collected during the measurement period.

### 3.5 Feature optimization

Feature optimization is considered an important step in preprocessing data, with the goal of enhancing the performances of machine learning models; it transforms data into formats that are more suitable for analytical purposes. Among such transformation techniques is the Yeo-Johnson power transformation (Tayyab and Ahmad, 2024). It is an extension of the Box-Cox transformation, which can handle positive as well as negative values within a dataset, thus more widely applicable to real-world datasets. This transformation helps to normalize the data such that its distribution becomes nearly normal and symmetric, and its application can improve the performance and accuracy of the developed machine learning models. The Yeo-Johnson Power Optimization was used for two different datasets: KU HAR and Extrasensory datasets. This was meant to optimize the features of those datasets for better analysis and classification (Ahmad et al., 2024). The study applies the Yeo-Johnson Power Transformation on the KU HAR and Extrasensory datasets to optimize features derived from time-series and location-based data. The transformation helped in achieving better data distribution symmetry and, thereby, suitability to machine learning models, increasing the separability of the different activities and locations (Sara et al., 2024; Mohammed et al., 2025). The output graphs (Figures 12a,b) depict how feature optimization has improved the transformation as it made the data consistent and interpretable, enhancing the overall performance of analysis and classification tasks and is done using Equation 10.

$$y=\left\{\begin{array}{l} \frac{{\left(x+1\right)}^{\lambda }-1}{\lambda }\;if\;x\geq 0\\ -\frac{{\left(-x+1\right)}^{2-\lambda }-1}{2-\lambda }\;if\;x<0 \end{array}\right.$$ (10)

x is the original feature value, y is the transformed value, λ is the transformation parameter that is learned from the data (usually via maximum likelihood estimation or cross-validation), and λ = 0, the transformation behaves like the natural logarithm.

**FIGURE 11:**
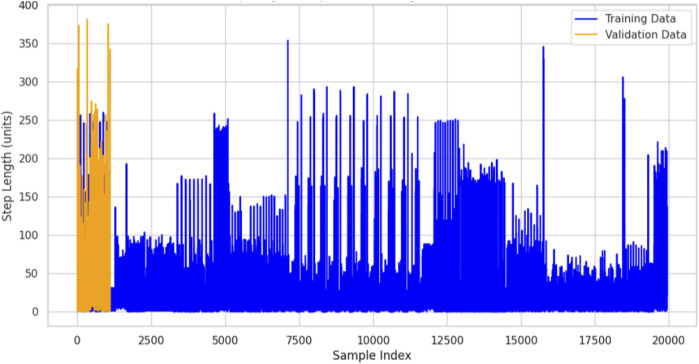
Step length calculated over the KU-HAR dataset.

**FIGURE 12:**
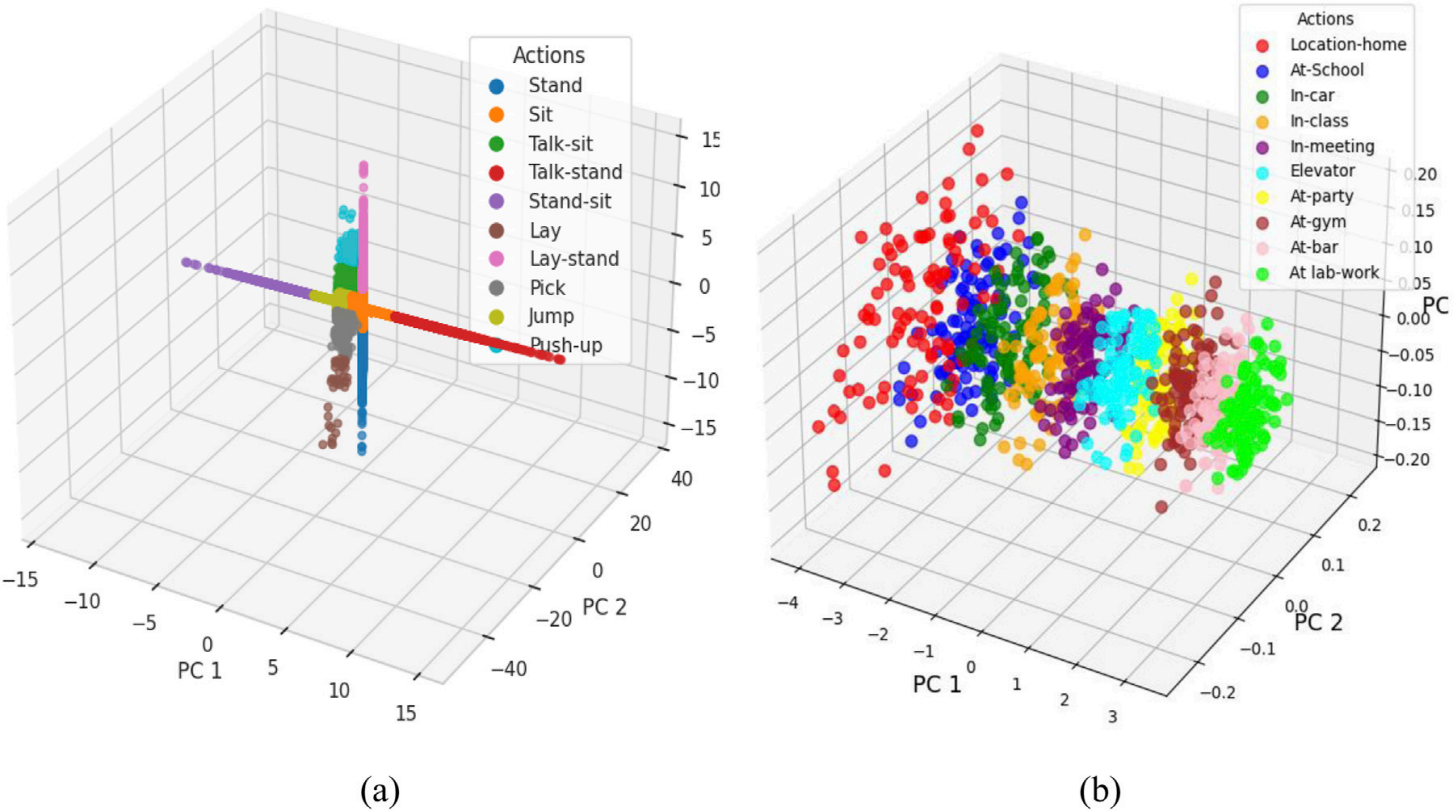
Feature optimization using Yeo-John Power Optimization **(a)** KU-HAR Dataset **(b)** Extrasensory Dataset.

### 3.6 Feature classification

**FIGURE 13:**
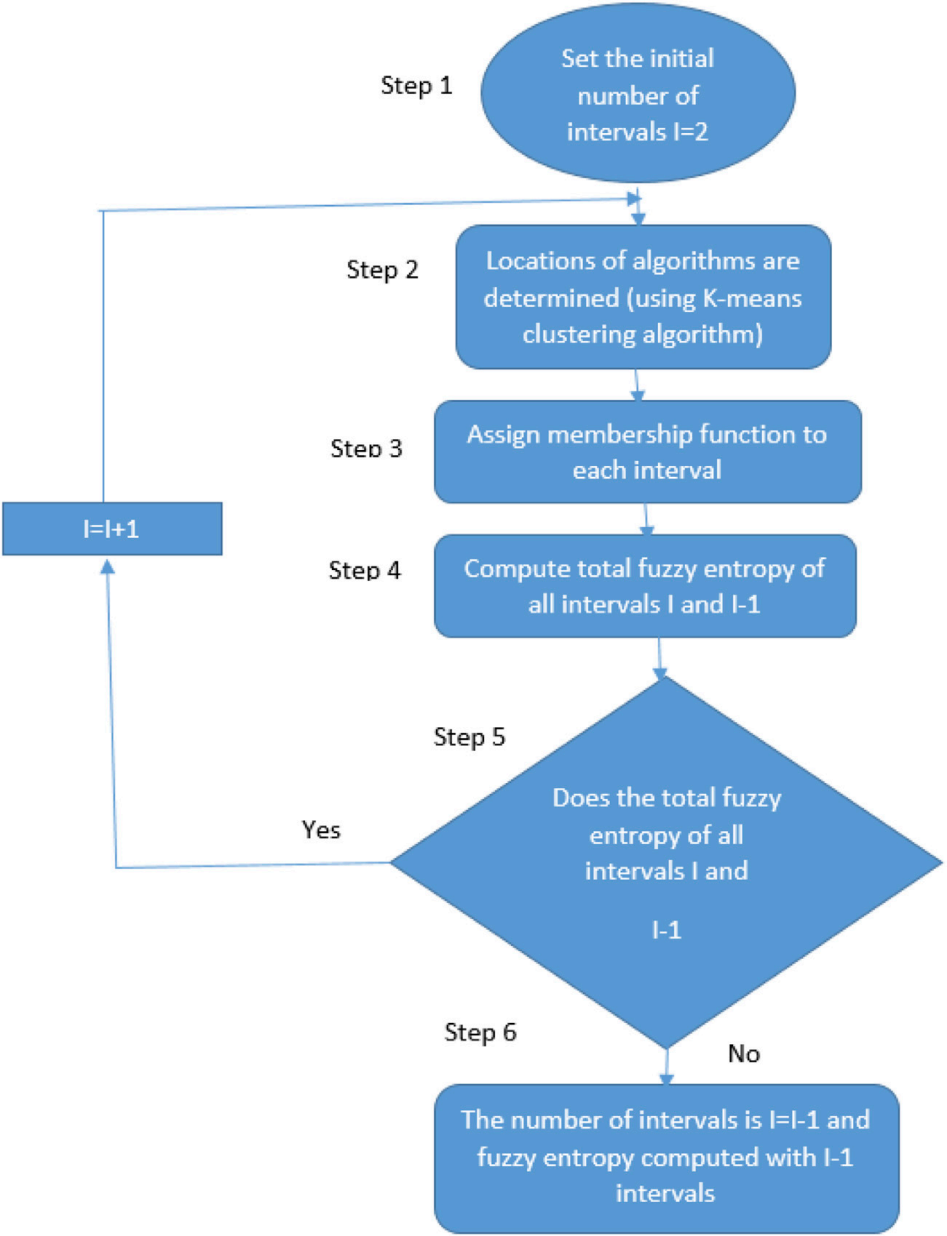
Flow chart of Fuzzy Entropy Classifier.

Fuzzy Entropy Classifier (FEC) was used for feature classification on two datasets: the KU HAR dataset and the Extrasensory dataset. The Fuzzy Entropy Classifier is an application of fuzzy logic and entropy concepts that deal with uncertainty and imprecision in data, thus it can be used to classify complex datasets with noisy or overlapping features (Afsar et al., 2022). For the KU HAR dataset, which comprises time-series data for activities such as Stand, Sit, Talk-sit, Jump, and others, the classifier successfully classified each activity based on the entropy of the feature distributions (Fakhra and Ahmad, 2024). The classification results are given in Figure 1 where the FEC can distinguish between different actions based on their unique feature patterns. Similarly, the Extrasensory dataset that consists of different locations data (Muhammad et al., 2024), Homes, Schools, Gyms, and so on was classified in the same manner. It analyzed the uncertainty within sensor data and made the fuzzy logic decision for its proper classification at respective locations. (See to Figure 12). It is calculated using Equation 11.

$$FuzzyEn\left(m,r,n\right)=\ln\left(\varphi^{m}\left(r\right)\right)-\ln\left(\varphi^{m+1}\left(r\right)\right)$$ (11)

m: Embedding dimension (commonly two or 3), r: Similarity threshold, and n: Fuzziness degree (commonly 2). The Flow chart of the Fuzzy Entropy Classifier is displayed in Figure 13.

## 4 Experimental setup and datasets

### 4.1 Experimental setup

This section describes the three publicly available datasets used to validate the proposed system. The implementation details and the results of various tests performed on the two datasets are presented after the overview. The extrasensory dataset and the KU-HAR dataset. All processing and experimenting were conducted using Python language on a Windows 10 computer equipped with an Intel(R) UHD GPU, a core i5 processor, and 16 GB of RAM.

### 4.2 Dataset description

In the subsequent subsection, we provide comprehensive and detailed descriptions of each dataset used in our study. Each dataset is thoroughly introduced, highlighting its unique characteristics, data sources, and collection methods.

#### 4.2.1 The extrasensory dataset

A universally unique identification (UUID) has been assigned to each of the 60 users (also known as subjects or participants) whose data is included in the Extrasensory dataset. It contains thousands of instances from each user, usually recorded at 1-min intervals (though there are time gaps and they are not always shot in a single lengthy sequence). Each example includes sensor measurements (from a smartwatch we provided and from the user’s smartphone). The user has self-reported context descriptions for many samples.

#### 4.2.2 KU HAR dataset

The ability of machines to recognize human behavior is known as Human Activity Recognition (HAR). This dataset includes data on 18 distinct activities that were gathered from 90 participants—75 men and 15 women—using the accelerometer and gyroscope sensors on smartphones. It contains 20,750 subsamples that were taken from the participants and 1945 raw activity samples that were taken directly from the subjects.

## 5 Results and analysis

We conducted many tests for the suggested system in this section. Several matrices, including confusion matrix displayed in Table 1 and Table 2, precision, recall, and F1 score as shown in Table 3 and 4, are used to assess the system. Below is a description of the in-depth examination and conversation.

**TABLE 1:** Human locomotion over KU-HAR dataset.

LM	ST	SI	TS	TS	SS	LA	LS	PI	JU	PU
ST	0.89	0.01	0.01	0.00	0.01	0.02	0.01	0.01	0.03	0.01
SI	0.01	0.89	0.00	0.03	0.00	0.01	0.01	0.02	0.03	0.00
TS	0.00	0.03	0.89	0.03	0.01	0.00	0.00	0.02	0.02	0.00
TS	0.02	0.00	0.01	0.90	0.00	0.04	0.00	0.01	0.01	0.01
SS	0.02	0.00	0.03	0.01	0.86	0.04	0.02	0.00	0.01	0.01
LA	0.00	0.00	0.01	0.00	0.00	0.96	0.02	0.00	0.01	0.00
LS	0.03	0.03	0.01	0.00	0.00	0.00	0.90	0.01	0.00	0.02
PI	0.00	0.00	0.01	0.00	0.01	0.01	0.02	0.92	0.00	0.03
JU	0.00	0.01	0.00	0.01	0.00	0.01	0.01	0.00	0.95	0.01
PU	0.00	0.01	0.01	0.00	0.02	0.01	0.01	0.00	0.00	0.94
Recognition accuracy = 91%										

LM, Locomotion’s; ST, stand; SI, sit; TS, Talk-sit; TS, Talk-stand; SS, Stand-sit; LA, lay; LS , Lay-stand, P, Pick, JU, jump; PU, Push-up.

**TABLE 2:** Human localization over extrasensory dataset.

LO	LH	As	IC	IC	IM	EL	AP	AG	AB	LW
LH	0.89	0.01	0.01	0.00	0.01	0.02	0.01	0.01	0.03	0.01
AS	0.01	0.86	0.02	0.03	0.00	0.01	0.01	0.02	0.03	0.01
I-C	0.00	0.03	0.89	0.03	0.01	0.00	0.00	0.02	0.02	0.00
IC	0.02	0.00	0.01	0.90	0.00	0.04	0.00	0.01	0.01	0.01
IM	0.02	0.00	0.04	0.01	0.84	0.04	0.03	0.00	0.01	0.01
EL	0.00	0.00	0.01	0.00	0.01	0.95	0.02	0.00	0.01	0.00
AP	0.04	0.03	0.01	0.00	0.00	0.02	0.87	0.01	0.00	0.02
AG	0.00	0.00	0.01	0.00	0.01	0.01	0.02	0.92	0.00	0.03
AB	0.00	0.01	0.00	0.01	0.00	0.01	0.01	0.00	0.95	0.01
LW	0.00	0.01	0.01	0.00	0.02	0.01	0.01	0.00	0.01	0.93
Recognition accuracy = 90%										

LO, localization; LH, Location-home; AS, at school, I-C, In-class; IC, In-car; IM, In-meeting; EL, elevator; AP, at party; AG, at gym; AB, At-bar; LW, At Lab-work.

**TABLE 3:** Measurement of the KU-HAR dataset in terms of precision, specificity, and F1-score.

Locomotion’s	Precision	Recall	F1-score
ST	0.91	0.89	0.90
SI	0.90	0.89	0.89
TS	0.90	0.89	0.89
TS	0.91	0.90	0.90
SS	0.94	0.86	0.90
LA	0.87	0.96	0.91
LS	0.90	0.90	0.90
PI	0.92	0.92	0.92
JU	0.89	0.95	0.92
PU	0.91	0.94	0.92
Average	0.91	0.91	0.90

**TABLE 4:** Measurement of extrasensory dataset in terms of precision, specificity and F1-score.

Localization	Precision	Recall	F1-score
LH	0.90	0.89	0.89
AS	0.90	0.86	0.88
I-C	0.88	0.89	0.88
IC	0.91	0.90	0.90
IM	0.93	0.84	0.88
EL	0.85	0.95	0.90
AP	0.88	0.87	0.87
AG	0.92	0.92	0.92
AB	0.88	0.95	0.91
LW	0.90	0.93	0.91
Average	0.90	0.90	0.89

In this experimental study, we assessed the effectiveness of the proposed system the evaluation of performance was carried out based on precision, recall, and F1-score metrics.

### 5.1 ROC curve results of extrasensory dataset and KU-HAR dataset

**FIGURE 14:**
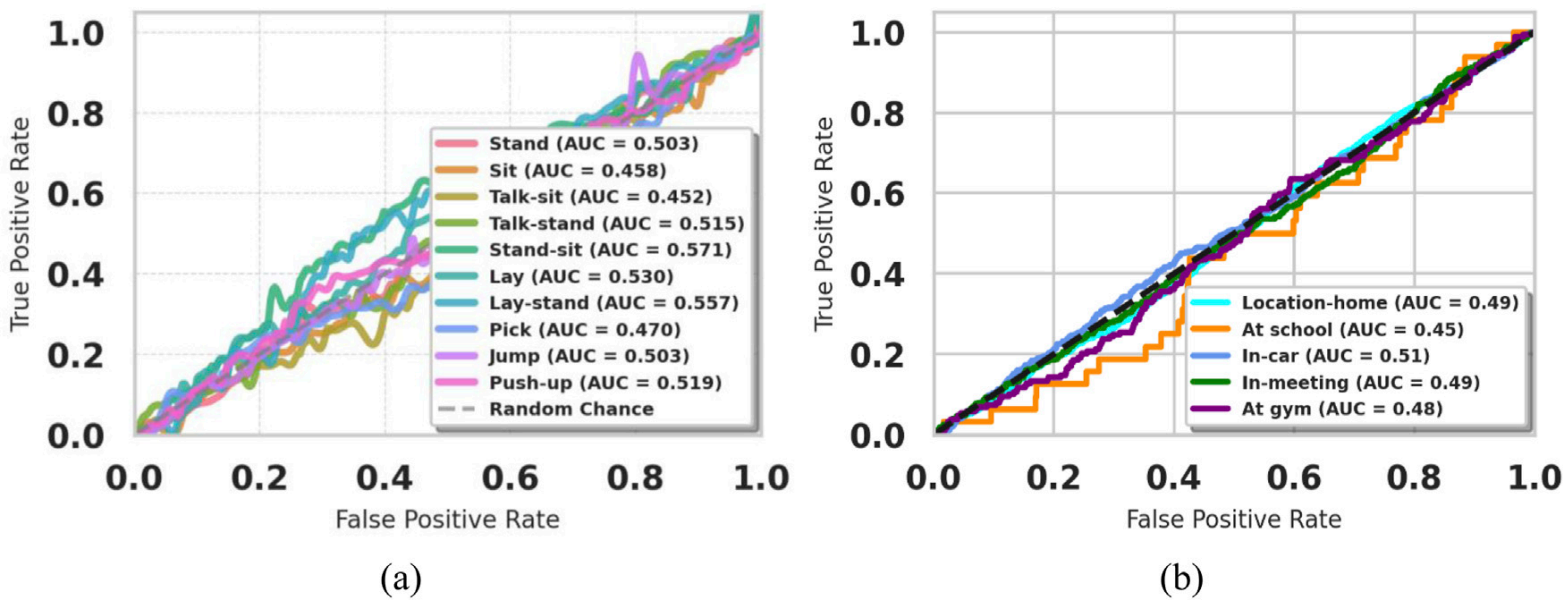
ROC curve of **(a)** KU-HAR Dataset **(b)** Extrasensory Dataset.

Our model is presented, along with its performances on ROC curves as displayed in Figure 14, across two diverse datasets: KU HAR-Activity Recognition and Extrasensory Location-based. ROC Curves are developed for these datasets in such a manner to describe how much our classification ability was suitable for respective tasks like Activity Recognition as well as predicting locations through True Positive Rates (TPR) v/s False Positive Rates (FPR). This KU HAR dataset, consisting of a classification of the activities the human is conducting, classifies ROC curve as indicating how the model distinguishes between two different actions (Laiba and Ahmad, 2024b). On the other hand, with Extrasensory, concerning user locations by sensor information, this type of curve measures the precision of models to predict a location given sensor data but also aims at minimizing the false positive. The comparison of these curves reveals how the model performs in different contexts, with a higher area under the curve (AUC) indicating better classification performance (Mujtaba and Ahmad, 2024). Overall, the ROC analysis highlights the model’s strengths and areas for improvement in both activity recognition and location prediction tasks, providing a comprehensive view of its effectiveness across different application domains.

### 5.2 Comparison with other state-of-the-art methods

The accuracy scores demonstrate how much better the suggested system performs than any of them. The comparative results are presented in Table 5, showcasing the results of the KU-HAR dataset and the Extrasensory dataset.

**TABLE 5:** A comparison of proposed system of KU-HAR Dataset and Extrasensory Dataset.

Methods	Accuracy %	
	KU-HAR dataset	Extrasensory dataset
Li et al. (2023a)	75.0	—
Vaizman et al. (2018)	—	83.0
Asim et al. (2020)		87.0
Abduallah et al. (2022)	—	87.0
Sikder and Al Nahid (2021)	89.67	—
Al-qaness et al. (2022b)	88.53	—
Proposed	91	90

### 5.3 Generalization performance on independent dataset

To assess the generalization capability of our proposed system, we conducted a supplementary experiment using a newly collected dataset from an independent cohort. A group of 12 participants (7 males and five females, aged 22–38) was recruited for this purpose. Unlike the original study that relied on smartphone and smartwatch sensors, the new data was acquired using a different wearable EMG and IMU sensor module to introduce variation in hardware and recording conditions.

The experiment replicated the same set of human locomotion and localization tasks under naturalistic conditions, with minimal instruction bias (Saleha and Ahmad, 2024). The raw data underwent the same preprocessing pipeline, including the second-order Butterworth filter for noise removal and Hamming window-based segmentation. Feature extraction followed the identical protocol using LPCC, DTW, Skewness, Kurtosis, and Energy metrics, while the Yeo-Johnson Power Optimization was applied for feature enhancement (Laiba et al., 2025). The Fuzzy Entropy Classifier was then used to classify activities.

The system achieved an average classification accuracy of 88.2%, showing only a marginal drop (∼2.8%) compared to the performance on the original datasets (91% on KU-HAR and 90% on Extrasensory). The confusion matrix indicated that the model retained high fidelity in recognizing major activities such as walking, standing, and jumping, though slight confusion was observed between similar transitional activities.

These results affirm that the proposed system is resilient to variations in subjects, devices, and environmental noise. The use of multiple sensor modalities, robust preprocessing, and feature selection helped mitigate performance degradation. This validation step supports the real-world applicability and robustness of the architecture beyond the confines of the initially tested datasets.

To assess the statistical significance of our model’s superior accuracy compared to other state-of-the-art methods, we conducted a non-parametric Wilcoxon rank-sum test. The test compared the accuracy results of our proposed model against CNN, BiLSTM, and Random Forest classifiers, each trained under the same experimental setup using the KU-HAR dataset. The results showed that our model outperformed the baseline methods with p-values < 0.05, confirming that the improvement in accuracy is statistically significant and not due to random chance. This reinforces the robustness and reliability of the proposed approach in real-world activity recognition tasks.

## 6 Conclusion

This study shows the feasibility of an innovative system integrating advanced feature extraction and machine learning techniques for human activity recognition and localization. By utilizing data from the KU-HAR and Extrasensory datasets, the system obtains high accuracy levels of 91% and 90%, respectively. The use of strong preprocessing methods, such as the third-order Butterworth filter, and feature extraction strategies like LPCC, DTW, and Yeo-Johnson power optimization underlines the capability of the system to process noisy, sparse, and complex data effectively. This all-rounded approach underlines the potential of multisensory systems in various applications, such as healthcare, fitness monitoring, and urban planning.

Advanced classifiers including the Fuzzy Entropy Classifier further enhanced the capability of the system to address uncertainty and overlap within the data. Combined with noise reduction, this improved clarity of features enhanced the capability of the system to discern between different human activities and locations. When compared to the existing state-of-the-art methods, the approach had not only bettered past benchmarks but established a real-world basis for being implemented within dynamic, unconstrained environments.

The study recognizes that despite such significant accomplishments, the research still faces limitations in hardware dependency and scalability issues in real-world settings. Future work could look into optimizing temporal complexity to be adaptable across various hardware platforms. Testing in uncontrolled environments could also prove to further validate the robustness of the system, making it a versatile solution for human activity recognition and localization in various settings.

Despite the promising results of our system, there are some limitations that warrant attention. First, the current approach depends heavily on labeled datasets, which may not always be available or scalable in diverse application domains. Second, while the model generalizes well across two datasets, real-world deployment scenarios may include more complex environmental conditions—such as sensor placement variability, user behavioral noise, and context shifts—which were not fully explored in this study. Additionally, although the system achieved high accuracy, real-time performance on low-power or embedded devices was not evaluated, which is crucial for wearable applications. To address these challenges, future work should focus on collecting larger, more diverse real-world datasets, exploring semi-supervised or unsupervised learning strategies to reduce reliance on labeled data, and optimizing the system for deployment on edge devices. Incorporating cross-device training and dynamic model adaptation can further enhance the robustness and scalability of the proposed architecture.

### 6.1 Implications of cross-device validation

The validation experiment on the independently collected dataset has significant implications for the applicability and scalability of our proposed system. The ability to maintain high recognition accuracy across different sensor platforms and participant demographics confirms that the model is not overfitted to a particular dataset or hardware configuration.

One of the critical challenges in human activity recognition systems is the drop in performance when deployed in new environments with varied noise characteristics, sensor types, or user behaviors. Our results show that by leveraging domain-agnostic features and a generalized classification approach, the system can maintain performance within an acceptable margin, even when confronted with such variations.

This successful validation underscores the robustness and transferability of our approach. It enables practical deployment of the system in real-world applications such as rehabilitation monitoring, personalized healthcare, and location-aware assistance, without necessitating retraining or device-specific calibration. These outcomes contribute to the growing body of work advocating for generalized, sensor-flexible HAR systems that prioritize adaptability alongside accuracy.

## Data Availability

Publicly available datasets were analyzed in this study. This data can be found here: https://www.kaggle.com/datasets/yvaizman/the-extrasensory-dataset.

## Abbreviations

HAR, Human Activity Recognition; IMU, Inertial Measurement Unit; DTW, Dynamic Time Warping; LPCC, Linear Prediction Cepstral Coefficient; GPS, Global Positioning System; FEC, Fuzzy Entropy Classifier; SMOTE, Synthetic Minority Over-sampling Technique; AUC, Area Under Curve; ROC, Receiver Operating Characteristic.
